# Incidence and determinants of excessive weight gain in people living with HIV initiating tenofovir, lamivudine, and dolutegravir-based therapy: a multicenter retrospective study in northwest Ethiopia

**DOI:** 10.3389/fphar.2025.1394458

**Published:** 2025-04-04

**Authors:** Gashaw Sisay Chanie, Wagaye Atalay, Tirsit Ketsela Zeleke, Zemenu Wube Bayleyegn, Yonas Sisay Aragie, Gizachew Kassahun Bizuneh, Mihret Melese, Rahel Belete Abebe

**Affiliations:** ^1^ Department of Clinical Pharmacy, School of Pharmacy, College of Medicine and Health Sciences, University of Gondar, Gondar, Ethiopia; ^2^ Department of Pharmacy, College of Health Sciences, Debre Markos University, Debre Markos, Ethiopia; ^3^ Department of Social and Administrative Pharmacy, School of Pharmacy, College of Medicine and Health Sciences, University of Gondar, Gondar, Ethiopia; ^4^ Department of Pharmacognosy, School of Pharmacy, College of Medicine and Health Sciences, University of Gondar, Gondar, Ethiopia; ^5^ Department of Human Physiology, School of Medicine, College of Medicine and Health 11 Sciences, University of Gondar, Gondar, Ethiopia

**Keywords:** excessive weight gain, tenofovir, lamivudine, and dolutegravir, PLHIV

## Abstract

**Background:**

The incidence and nature of excessive weight gain associated with antiretroviral treatment using tenofovir, lamivudine, and dolutegravir based regimens among patients living with human immunodeficiency virus has not been properly examined in Ethiopia. Therefore, this study aimed to assess the incidence and factors associated with excessive weight gain among People living with human immunodeficiency virus on tenofovir, lamivudine, and dolutegravir based regimens in a real-world setting.

**Method:**

A multicenter retrospective cross-sectional study was conducted from December 1, 2022, to August 30, 2023, involving 620 human immunodeficiency virus patients initiating a tenofovir, lamivudine, and dolutegravir based regimen. Data on sociodemographic, clinical details, and excessive weight gain were collected from medical records and patient interviews using a semi-structured questionnaire. Continuous variables were reported with mean and standard deviation. Binary logistic regression analysis was performed, and variables with a P-value ≤0.25 were included in multivariate logistic regression. Statistical significance was set at a P-value of ≤0.05.

**Results:**

A total of 620 participants were involved in the analysis, revealing a 31.43% incidence of excessive weight gain 95%CI (27.1–36.0). The mean weight gain was 3.77 kg with a 1.5 SD at 72 months follow-up. Factors such as being female [AOR = 1.75, 95% CI (1.01, 3.04)], age between 38–46 years [AOR = 1.53, 95% CI (1.23, 2.76)], lack of physical activity were [AOR = 4.41, 95% CI (1.46, 11.80)], having 6–12 months and 13–24 months of since starting new regimen follow up duration [AOR = 3.35, 95% CI (2.79, 4.30)] and [AOR = 2.67, 95% CI (2.43, 3.25)] respectively and having detectable viral load at initiation of regimen [AOR = 2.34, 95% CI (1.18, 6.63)] were significantly associated with excessive weight gain.

**Conclusion:**

PLHIV receiving a tenofovir, lamivudine, and dolutegravir based regimen particularly females, aged 38–54 years, those with limited physical activity, follow-up durations of 6–24 months, advanced disease stages, and a detectable viral load at therapy initiation should be closely monitored for weight gain. Proactive surveillance in these patient groups is crucial to optimize therapeutic outcomes and address potential health concerns associated with weight changes.

## Introduction

The development of antiretroviral therapy in the last decades provided a long-term viral suppression that translates into higher quality of life, longer life expectancy, and an impressive decrease in HIV-related morbidity and mortality for People Living with HIV (PLHIV) (Cardoso-Neto et al., 2023; Kanters et al., 2016).

There is a growing body of kinds of literature that the use of modern ART regimens such as integrase strand transfer inhibitor (INSTI) based ART, Dolutegravir (DTG) in particular leads to a statistically significant increase in body weight even clinical obesity. This effect seems to be more pronounced in those with more pronounced CD4^+^ cell count depletion, black people, and women (Hill et al., 2019; Koethe et al., 2016; Thivalapill et al., 2021a). More recent reports from both real-world and randomized clinical trial settings suggest that integrase strand transfer inhibitor (INSTI) based ART use may be associated with excess weight gain, particularly when compared with the use of nonnucleoside reverse transcriptase inhibitor (NNRTI)based ART (Bourgi et al., 2020a; Brennan et al., 2023; Kanters et al., 2022).

According to Clinton’s healthcare estimation, more than 90% of patients living with HIV in low- and middle-income countries are taking dolutegravir-containing fixed-dose combinations which suggest that dolutegravir improve patient outcomes and ultimately lead to cost savings (Brief, 2019)**.**

The World Health Organization (WHO) and the Ethiopia national guideline recently recommended using INSTIs, specifically dolutegravir (DTG) either with tenofovir disoproxil fumarate (TDF) and lamivudine (3TC) (TDF + 3TC + DTG) based regimen as a preferred first-line antiretroviral therapy (ART) and DTG is replaced by efavirenz (EFV) because of dolutegravir effectively controls viral replication, barrier to resistance and safety benefits over efavirenz (Brief, 2019; Balaji et al., 2024). However, despite its efficacy, there is evidence of a new side effect that has emerged in recent years an excessive weight increase associated with the use of TDF + 3TC + DTG -based regimens among treatment naïve and experienced patients (Cardoso-Neto et al., 2023; Koethe et al., 2016; Brennan et al., 2023; Ando et al., 2021a; Caniglia et al., 2020; Kerchberger et al., 2020; Menard et al., 2017). Several recent studies have linked greater weight gain among persons receiving TDF + 3TC + DTG based ART regimens for initial therapy than other HIV drug classes (Bourgi et al., 2020a; Kanters et al., 2022).

A recent network meta-analysis showed that dolutegravir (DTG) in combination with Tenofovir (TDF) and lamivudine (3TC) was associated with a higher increase in weight gain compared with regimens that used DTG with other Nucleoside Reverse Transcriptase Inhibitors (NRTI) as a backbone (Kanters et al., 2022).

Weight gain after ART initiation was considered favorably regarded as a health return phenomenon earlier but more recently several concerns about long-term ART-associated excess weight gain (high BMI) with DTG and its associated cardiometabolic complications, such as cardiovascular disease, diabetes, hypertension, neurocognitive impairment, and other comorbid conditions, and the avoidance of weight gain may reduce these risks (Bosch et al., 2023).

A previously published analysis on the 5- and 10-year risks of cardiovascular disease and diabetes was conducted in Kenya. This demonstrated that participants on the TDF + 3TC + DTG based regimen had significantly greater risk scores for development of cardiovascular disease or diabetes, driven by weight gain (McCann et al., 2021). Similarly a prior cohort study in Kenya showed that people with HIV (PLHIV) starting TDF + 3TC + DTG based regimen were at significantly higher risk for weight gain (Bourgi et al., 2023). Numerous studies have additionally emphasized that a specific subset of individuals initiating INSTI (African decent, those with lower body weight, and people on tenofovir as a companion drug to DTG) are at risk for greater weight gain (Bourgi et al., 2020a; Ando et al., 2021a; Lake et al., 2020).

A higher pre-ART treatment HIV viral load, female gender, black race, and older persons were experienced greater weight gain (Hill et al., 2019; Bourgi et al., 2020a; Brennan et al., 2023; Lake et al., 2020; Sax et al., 2020). Baseline CD4^+^ count ˂200cells/mm^3^, being ART-naïve and being on ART ≥24 months were also associated with higher weight change from baseline (Cardoso-Neto et al., 2023; Koethe et al., 2016; Brennan et al., 2023; Sax et al., 2020; Taramasso et al., 2020). In contrast female gender were represented factor that was protective against becoming weight gain seen in one observational cohort studies conducted in Italy (Taramasso et al., 2020).

There is a limited data concerning the effect of TDF + 3TC + DTG based regimen ART on body weight in the Ethiopian population. Given the clinical implications of higher body weight on long term health and the broad adoption of TDF + 3TC + DTG based regimens as a recommended first-line therapy; we needed to conduct a research on one of the first-line TDF + 3TC + DTG based regimen “Incidence and determinate factors associated of weight gain with TDF + 3TC + DTG based regimen in PLHIV at North-west comprehensive specialized hospital ART follow up clinics. This study was a part of the project “Patients reported neuropsychiatric adverse effects initiating TDF + 3TC + DTG -based regimen Antiretroviral Therapy among adults living with HIV in real-life clinical practice Ethiopia”.

To the best of our knowledge, this is the first study to investigate the effect of TDF + 3TC + DTG based regimen ART on excessive body weight gain in Ethiopian people living with HIV. Hence, our preliminary finding incites the need to monitor the effect of TDF + 3TC + DTG based regimen on excessive body weight gain.

## Methods

### Study design, setting and study period

PLWH who began their first TDF + 3TC + DTG based regimen between December 1, 2022, and August 30, 2023, were included in a multicenter retrospective with cross-sectional study. The study was conducted at multi-facility public compressive specialized hospitals (CSHs) of ART follow up clinics found in Amhara region, Ethiopia. The three CSHs were Felegehiowt, Debre Tabor CSH, and Gondar CSH were selected by lottery method out of the total of eight CSHs found in Amhara regional state. These are far away 492–727 km from Addis Ababa. More over 2.5 million, seven million, and five million residents of the catchment region are served by the hospitals, respectively (Zemariam et al., 2023). It is 37.5% of the total CSHs which is statistically appropriate according to WHO criteria (>30%). The region was selected due to various socio cultural experiences are found across the population which makes the study to be generalized. From January 2019 to August 2023 a total of 620 adult PLWH who were receiving TDF + 3TC + DTG based ART regimens at the selected CSHs were included.

### Inclusion criteria

All PLHIV who were at least 18 years of age, had been on first-line ART for at least 6 months and had started ART for the first time after Ethiopia implemented the WHO test-and-treat strategy 2018 (Ethiopia, 2018) were included. Patients who had started treatment with TDF + 3TC + DTG based regimen at the baseline and stayed on the same regimen for more than 72 months and had at least two of the consecutive weight measure data starting from the baseline were recorded.

### Sample size determination and sampling procedures

The sample size was calculated using single population proportion formula with the assumptions of 95% confidence interval (CI), marginal error (d) of 5%. Considering the proportion 50% for weight gain to take the maximum possible sample size, considering the design effect (two stage) and adding 10% non-response rate, the sample size was 846.

Option 2:

$$n=\frac{{\left(z_{\frac{a}{2}}\right)}^{2}\times p\times \left(1-p\right)}{d^{2}}=\frac{{\left(1.96\right)}^{2}\times 0.5\times \left(1-0.5\right)}{{\left(0.05\right)}^{2}}\;X\;2\;\left(\mathrm{design}\;\;effect\right)+10\%=846$$

**FIGURE 1:**
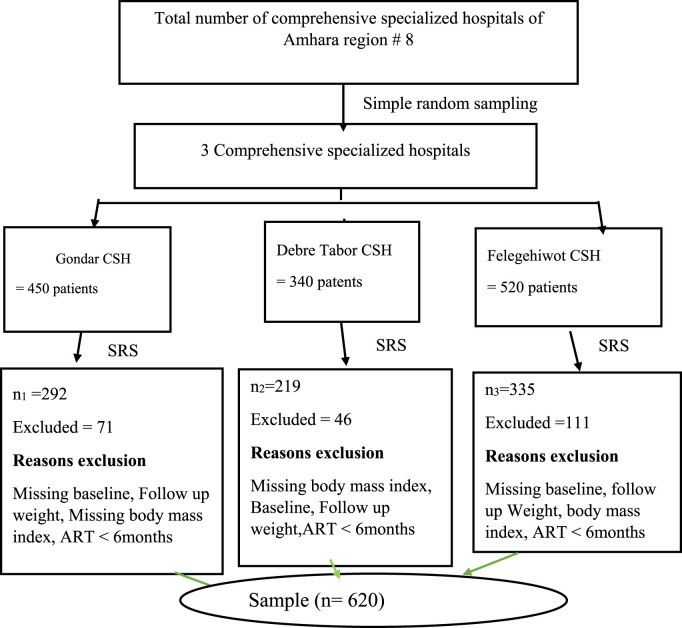
Schematic presentation of the sampling procedure for the assessment of weight gain to initiating TDF + 3TC + DTG based regimen among PLH patients, 2023 (n = 620).

The three CSHs, Felegehiwot CSH, Debre Tabor CSH, and Gondar were selected by lottery method out of a total of eight CSHs found in Amhara Regional State. It is 37.5% of the total CSHs, which is statistically appropriate according to WHO criteria (>30%). The number of adults PLH patients attending ART follow-up clinics initiating TDF + 3TC + DTG based regimen was taken from each selected hospital. The sample size was survey to each of the selected hospitals (Figure 1). A survey sampling technique was used from each selected hospital. The patient record charts was obtained from the patients during each follow-up in the hospital ART clinics.

### Data collection instrument

An interviewer-administered method of data collection with chart review on anthropometric measurements from ART chart as recorded by physician and ART clinic nurses were used. The collection tool had three sections: the first was sociodemographic characteristic completed through patient interviews and collected information on social demographics such as sex, age, marital status, religious affiliation, level of education and employment status, level of physical activities alcohol and khat use and the time the medicine is taken. The second section was baseline anthropometric and clinical characteristics of the study population characteristics completed by file reviewer, which included the duration since the start of TDF + 3TC + DTG based regimen ART, WHO clinical stage, and comorbidity, etc.

### Study variables

#### Dependent variable

The Incidence of excessive weight gain.

#### Independent variables

Sociodemographic characteristic (gender, age, residency, marital status, religion, employment status, level of education, alcohol use, smoking status, khat use, level of physical activity), Clinical and anthropometric measurements.

### Data collection and data quality assurance

Six data collectors with prior data collection experience were recruited, along with three professional supervisors, and data collectors were instructed to define the aim of the study. Each study setting had one supervisor and two data collectors. Supervisors implemented intensive follow-up in each study site. Participants in the study were contacted during their outpatient visits to the ART clinic at each CSH.

Data was collected through face-to-face interviews with study participants who provided informed consent. Data collecting technologies were tested at Dessie CSH ART clinic with 15 patients in each group to examine the completeness, correctness, and accuracy of the patients’ charts. On a daily basis, the data was checked for completeness and consistency. Each patient file’s data abstraction form was issued an identification code. The filled Forms were examined for accuracy, consistency, and completeness by the principal investigator. Completed forms were kept in a secure location, protecting patient confidentiality and data from tampering. The data gathering instrument was translated into Amharic, a widely spoken local language.

### Statistical analysis

Using Epi data 3.0, all completed data collecting forms were reviewed, coded, and data entered. To detect any errors, data cleaning and validation were performed. The data was exported and processed with SPSS version 25.0, a statistical software for social sciences. Descriptive statistics were used to present socio-demographic, clinical characteristics whereas frequencies and percentages were used to present categorical data. Text, tables, and graphs were used to present the data. For continuous variables, paired t-tests with mean values and 95% confidence intervals (CI) were used as summary anthropometric measurements to determine excessive weight gain. Bivariate analysis was used to establish the association between parameters related with excessive weight gain and the TDF + 3TC + DTG based regimen.

The multivariable analysis included all factors with a p-value <0.25 from the bivariate analysis. P-value with adjusted odds ratio at 95% confidence interval was less than 0.05, the variable was considered statistically significant.

## Ethical considerations

The University of Gondar College of Medicine and Health Sciences School of Pharmacy Ethical approval Review Committee November 22, 2022, SOP (088/2022) granted ethics approval. After obtaining consent from each facility, the data was collected. Because the study was conducted through patient interviews and the analysis of medical records, no individual patients would be harmed as long as confidentiality was maintained. Information acquired from patients and medical records was kept strictly secret and utilized only for this study. This study complied with the Helsinki Declaration.

### Operational definition

Baseline weight was defined as the closest weight recorded 6 months for those in the TDF + 3TC + DTG based regimen (Siegmund et al., 2018).

Follow-up weight was defined 72 months after the start of follow up with the closest weight recorded 6 months prior to that 72 -month end point.

Change in weight was calculated by simply subtracting the mean of 72-month follow-up weight from baseline weight (Stevens et al., 2006).

Excessive weight gain we chose a weight gain of ≥10% as the end variable after 72 months of treatment follow-up (Bourgi et al., 2020b).

## Results

### Socio-demographic characteristics

In this study, six hundred twenty adult’s patients living with HIV on TDF + 3TC + DTG based regimen were included for the final analysis. Half of the study participants which two hundred and eleven (50.2%) were males. The mean age was 32.5 years (±10.42) which ranged from 18 to 76 years. A significant portion of the research participants, constituting 62.4%, were unemployed. Regarding physical activity, around 34.0% and 31.2% engaged in none and minimal physical activity respectively (Table 1).

**TABLE 1:** Sociodemographic characteristics of PLHIV on TDF + 3TC + DTG based regimen, Northwest Ethiopia (n = 620).

Variable	Variable	Frequency (N, %)
Gender	Male	313 (50.2)
	Female	307 (49.8)
Age	20–37 years	156 (25.2)
	38–46 years	189 (30.4)
	47–54 years	122 (19.6)
	55–75 years	154 (24.8)
Residency	Urban	485 (78.3)
	Rural	135 (21.7)
Marital status	Single	118 (19.0)
	Married	366 (59.0)
	Widowed	102 (16.4)
	Divorced	34 (5.5)
Religion	Orthodox	538 (86.7)
	Muslim	74 (11.9)
	Catholic	9 (1.4)
Employment status	Employed	233 (37.6)
	Unemployed	387 (62.4)
Level of Education	Unable to read and write	143 (23.1)
	Primary (Cardoso-Neto et al., 2023; Kanters et al., 2016; Hill et al., 2019; Koethe et al., 2016; Thivalapill et al., 2021a; Bourgi et al., 2020a; Brennan et al., 2023; Kanters et al., 2022)	161 (26.0)
	Secondary (Brief, 2019; Balaji et al., 2024; Ando et al., 2021a; Caniglia et al., 2020)	149 (24.0)
	college and above	167 (26.9)
Alcohol use	Yes	248 (39.8)
	No	372 (58.2)
Smoking status	Yes	30 (4.8)
	No	590 (95.0)
Khat use	Yes	37 (6.0)
	No	583 (94.0)
Level of physical activity	None	128 (7.1)
	Minimal	170 (27.6)
	Moderate	210 (34.0)
	Active	192 (31.2)

### Baseline anthropometric and clinical characteristics of study participants

More than one -third (36.3%) study participants had 13–24 months duration of TDF + 3TC + DTG based regimen ART follow up. About half (50.0%) of study participants had WHO clinical stage of III and IV. And (51.3%) of the study participants had detectable viral load during initiation of TDF + 3TC + DTG based regimen (Table 2).

**TABLE 2:** Baseline clinical and immunological profile of PLHIV on TDF + 3TC + DTG based regimen, Northwest Ethiopia (n = 620).

Variable	Variable	Frequency (N, %)	95% CI
Duration since the start of TDF + 3TC + DTGbased regimen ART drugs (month)	6–12 months	197 (31.8)	
	13–24 months	225 (36.3)	
	24–72 months	198 (31.9)	
WHO clinical stage	I	124 (20.0)	
	II	186 (30.0)	
	III and IV	310 (50.0)	
Comorbidity	Yes	216 (34.8)	
	No	404 (65.2)	
Type of comorbidity	Hypertension	135 (62.5)	
	Diabetic Mellitus	81 (37.5)	
Concurrent use of other medication	Yes	186 (29.8.0)	
	No	434 (70.2)	
Concurrent use of other medication	Anti-DM drugs	107 (57.6)	
	Antihypertensive drugs	79 (42.4)	
Type of opportunistic infection	TB	155 (25.2)	
	None	465 (74.8)	
Medication for Opportunistic infection	Yes	143 (92.3)	
	No	12 (7.7)	
Time of the day medication is taken	Morning	32 (5.2)	
	Night	578 (93.3)	
	Both	10 (1.4)	
Medication taking in relation to meal	Before meal	186 (30.4)	
	After meal	434 (69.6)	
Adherence for treatment	Poor	94 (15.2)	
	Fair	64 (10.2)	
	Good	461 (74.5)	
Anthropometric measurements	Baseline mean ± SD	Final mean ± SD	
Weight (kg)	54.65 ± 10.5	58.42 ± 11.5	3.21 (4.32–13.34)
BMI (kg/m^2^)	21.68 ± 4.86	23.18 ± 5.3	1.28 (1.73–13.02)

### Incidence of excessive weight gain among study participants

**FIGURE 2:**
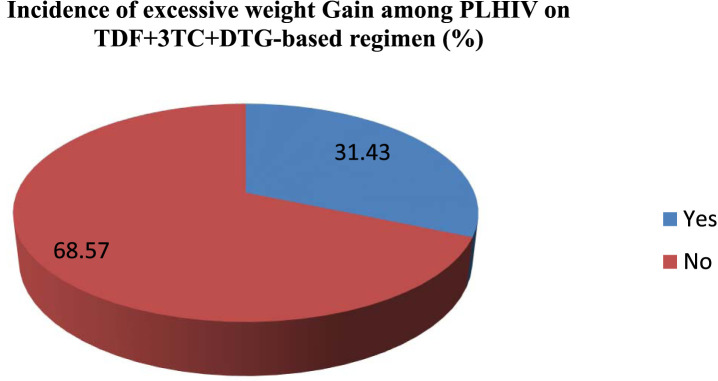
Incidence of excessive weight gain among PLHIV initiating TDF + 3TC + DTG based regimen northwest Amhara, Ethiopia hospitals (n = 620).

In the current study one hundred and ninety-five 31.43%, 95% CL (27.1–36.0) participants reported excessive weight gain (Figures 2).

### Incidence and determinant factors associated with excessive weight gain

In the current study three hundred and twenty-six (31.43%), 95% CI (27.1–36.0) study participants had excessive weight gain. In the current study, sex, age, employment status, level of physical activity, duration since the start of TDF + 3TC + DTG based regimen ART drug in months, WHO clinical stage and detectable viral load at initiation of TLD based regimen were candidate variables for the final model and entered into multivariable logistic regression. In the final model; being female [AOR = 1.75, 95% CI (1.01, 3.04)], age between 38–46 years [AOR = 1.53, 95% CI (1.23, 2.76)], none level of physical activity were [AOR = 4.41, 95%CI (1.46, 11.80)], having 6–12 months and 13–24 months of since starting TDF + 3TC + DTG based regimen follow up duration [AOR = 3.35, 95% CI (2.79, 4.30)] and [AOR = 2.67, 95% CI (2.43, 3.25)] respectively and having detectable viral load at initiation of TDF + 3TC + DTG based regimen [AOR = 2.34, 95% CI (1.18, 6.63)] were significantly associated with excessive weight gain (Table 3).

**TABLE 3:** Bivariate and multivariate analysis of determinant factors associated with excess weight gain among Person living with HIV on TDF + 3TC + DTG based regimen (n = 620).

Variable	Category	Excess weight gain		COR (95%CI)	AOR (95%CI)
		Yes	No		
sex	Male	77 (24.6%)	236 (75.4%)	1	1
	Female	118 (38.3%)	189 (61.7%)	1.9 (1.25–2.88)	1.75 (1.01, 3.04)^**^
Age	20–37 years	38 (20%)	118 (80%)	1	1
	38–46 years	60 (32%)	129 (68%)	1.88 (1.03–3.45)	1.53 (2.23, 2.76)**
	47–54 years	41 (33.7%)	81 (66.3%)	2.04 (1.05–3.94)	1.43 (2.01, 2.54)
	55–75 years	61 (40.4%)	93 (59.6%)	2.71 (1.46–5.03)	1.41 (0.76, 2.03)
Employment status	Employed	158 (67.7)	75 (32.3)	1	1
	Unemployed	316 (81.7)	71 (18.3)	2.13 (1.35, 3.35)	1.94 (0.87, 3.35)
Level of physical activity	None	77 (60%)	51 (40%)	4.84 (2.10–11.14)	4.14 (1.46, 11.80)**
	Minimal	67 (39.7%)	103 (60.3%)	2.12 (1.22–3.67)	1.75 (1.04, 2.94)
	Moderate	54 (25.9%)	156 (74.1%)	1.126 (0.65–1.95)	1.04 (0.98, 2.34)
	active	45 (23.7%)	147 (76.3%)	1	1
Duration since the start of ART drug	6–12 months	99 (50.3%)	98 (49.7%)	3.588 (2.04–6.32)	3.35 (2.79, 4.30)*
	13–24 months	114 (50.7%)	111 (64.4%)	2.581 (1.48–4.51)	2.67 (2.43, 3.25)**
	24–72 months	33 (17%)	165 (83%)	1	1
WHO clinical stage	I	34 (27.0%)	90 (73.0%)	1	1
	II	107 (57.5%)	79 (42.5%)	4.47 (1.76–11.45)	4.2 (1.65–9.75)*
	III and IV	176 (57.2%)	134 (42.8)	4.00 (1.50–10.40)	4.5 (2.65–11.78)**
Viral load at initiation	Detectable	296 (88.0%)	40 (12.0%)	3.24 (2.47–7.35)	2.34 (1.18–6.63)**
	Undetectable	99 (35.0%)	185 (65.0%)	1	1

Hosmer and Lemeshow goodness of fit p = 0.741, *p < 0.05 and **p < 0.01.

## Discussion

Current World Health Organization and Ethiopia national comprehensive guidelines recommend to use TDF + 3TC + DTG based combinations as preferred first- and second-line ART regimens in for ART because of high efficacy (Brief, 2019; Balaji et al., 2024).

The goal of this study was to determine the incidence and its determinant factors associated with excessive weight gain among adult patients living with HIV who is on TDF + 3TC + DTG based regimen at comprehensive specialized hospitals at Amhara Northwest Ethiopia.

### Incidence of excessive weight gain

The study participants had a high incidence of excessive weight gain 31.43%, 95% CI (27.1–36.0). Which is consistent with a prior study conducted in US in which a substantial proportion of participants after initiation of TDF + 3TC + DTG based regimen had become excessive weight gain (32%) (Bourgi et al., 2020a), Eswatini which was 35.3% (Mukuna et al., 2024). However this findings is higher than another earlier study conducted in US, and Botswana which 22%, and 12.9% of participants had excessive weight gain after TDF + 3TC + DTG based regimen treatment initiation (Caniglia et al., 2020; Kerchberger et al., 2020). The possible reason for discrepancies in prevalence rates of weight gain in this TDF + 3TC + DTG based regimen could be explained by differences in sociodemographic characteristics, sample size, and study settings.

This study further showed that patients received TDF + 3TC + DTG based regimen the mean weight gain was 3.76 kg compared with baseline during the 72 months follow-up. A similar study done in France among patients initiating TDF + 3TC + DTG based regimen had abnormal weight gain, which ranged between 4 and 12 kg (Menard et al., 2017), in Southern Ethiopia, participants who started TDF + 3TC + DTG based regimen a mean weight gain of 3.88 kg, 8.6 kg (Hirigo et al., 2023; Kure et al., 2022). Similarly participants who starting TDF + 3TC + DTG based regimen weight gain with a mean of 1.28 kg/m^2^ (1.73–13.02) at 72 months follow up compared to baseline. Long-term treatment may lead to increased risk of metabolic and cardiovascular disease in this population. A study found that a 1 kg/m2 rise in BMI after commencing ART increases the chance of developing diabetes and cardiovascular disease by 12% and 18%–20%, respectively, regardless of pre-ART BMI (Achhra et al., 2016). A study conducted by Veterans Affairs found that PLHIV had a 14% higher chance of getting diabetes mellitus when their weight increased by 5% compared to veterans without HIV infection (Herrin et al., 1999).

### Determinant factors associated with excessive weight gain

This study showed, female received TDF + 3TC + DTG based regimen first line ART regimen were much more likely to have a significant body weight gain than male. The data found in the current study is supported by most previous studies conducted in different US states, Brazil, Boston and Botswana, Uganda, and Ethiopia (Cardoso-Neto et al., 2023; Bourgi et al., 2020a; Caniglia et al., 2020; Bourgi et al., 2023; Lake et al., 2020; Sax et al., 2020; Thivalapill et al., 2021b; Esber et al., 2022; Alebel et al., 2022) In contrast, one study conducted in Italy (Taramasso et al., 2020), in Japan (Ando et al., 2021b) and Ethiopia (Weldesenbet et al., 2020) did not show this association, instead the report was that being female was a protective against excessive weight gain. Gender variations in weight growth may be linked to hormonal differences and a higher likelihood for females. HIV patients may experience anxiety and despair, which can have a significant impact on their body weight (Albert, 2015; Kuehner, 2017). Implies that gender-specific interventions and close follow-up are needed to improve weight among patients living with HIV on ART. This discrepancy could be due to the difference in the population characteristics included in the study and race. The other possibilities might be due to lifestyle characteristics of the study population.

This study observed a positive association between 38 and 54 years and weight gain. However, the association was not found beyond this age group, which is somehow inconsistent with previous studies conducted in US, in which the association was seen in older ages (Koethe et al., 2016; Lake et al., 2020). The result found in this study is slightly in line with a study from South Africa which age <50 years was a correlations with weight gain (Brennan et al., 2023). The possible explanation could be that older patients taking several complex medications to manage different health conditions may increase their chances of being prescribed drugs increase body weight or the other confounding were not properly managed.

This study found an association between baseline viral load and excessive weight in multivariate models; participants who had a pretreatment detectable viral load were associated with weight gain is further supported by a retrospective cohort studies conducted in US and South Africa (Koethe et al., 2016; Brennan et al., 2023; Sax et al., 2020). The association could be explained by return-to-health phenomenon to weight gain in PLWH initiating ART since the viral load is suppressed by ART. This effect may be desirable in some individuals, but could also contribute to excess weight gain in individuals with early-stage HIV disease and those with normal or above-normal BMI.

In this study patients on treatment 6–12 months and 13–24 months since starting of TDF + 3TC + DTG based regimen follow up were associated in increasing weight gain over the >24 months follow-up period. This study also showed, a significant association between none physical activity with increase body weight. However, the methodological diversity of the available studies makes difficult to define the role of each of these variables on the weight changes in real life world.

## Strengths and limitations

The primary strength of this study was that it was multicenter retrospective with cross-sectional, and it was the first to assess the prevalence of excessive weight gain and its determining factors in first-line therapy with a TDF + 3TC + DTG based regimen among adults in Ethiopia. This makes it a cornerstone for future research. This study employed physician-confirmed and patient-reported anthropometric measurements, as well as laboratory data, to illustrate the real-life scenario of significant weight gain associated with TDF + 3TC + DTG based regimens. We used a more than 5-year retrospective cross-sectional methodology to analyze excessive weight gain associated with TDF + 3TC + DTG based regimen treatment. However, the study has limitations, such as the fact that we only used descriptive statistics and that numerous variables were excluded due to incompleteness because the data source was secondary (chart review). Moreover our analysis did not adjust for exposure to non-HIV medications associated with weight gain (e.g., hormonal and psychotropic drugs) nor did we adjust for pregnancy, as these data were not routinely collected in the cohort. Finally, we did not assess clinical outcomes, such as metabolic or cardiovascular disease incidence or progression, and future studies will be needed to determine the impact of the observed weight changes on the health of PLHIV.

## Conclusion

In a real-world population, patients gained weight after beginning a TDF + 3TC + DTG based regimen. Longer prospective cohort follow-up and post market surveillance especially on pharmacovigilance are required to assess whether TDF + 3TC + DTG based regimen-related weight increase is connected with changes in non-communicable disease risk over time, or whether weight gain is sustained, as reported in clinical trials.

## Data Availability

The dataset used and analyzed during this study is available from the corresponding author upon reasonable request.

## Abbreviations

AOR, adjusted odds ratio; ART, antiretroviral treatment; BMI, body mass index; CI, confidence interval; CSH, compressive specialized hospitals; COR, crude odd ratio; DTG, dolutegravir; EFV, efavirenz; INSTI, integrase strand transfer inhibitor; NRTI, nucleoside reverse transcriptase inhibitors; 3TC, lamivudine; PLHIV, persons living with human immunodeficiency virus; TDF, tenofovir; WHO, World Health Organization.
